# Complete response of advanced rectal gastrointestinal stromal tumors after imatinib treatment: A case report and literature review

**DOI:** 10.1097/MD.0000000000029411

**Published:** 2022-08-12

**Authors:** Tingting Wu, Xiaobin Cheng, Wenbin Chen

**Affiliations:** a Department of Colorectal Surgery, The First Affiliated Hospital, Zhejiang University School of Medicine, Zhejiang, People’s Republic of China.

**Keywords:** advanced GIST, complete response, imatinib, rectal tumor

## Abstract

**Rationale:**

Patients with rectal gastrointestinal stromal tumors (GISTs) who achieve a complete response (CR) with imatinib therapy have rarely been reported in the literature. Moreover, no treatment guidelines have been established for rectal GIST patients with CR after imatinib treatment, warranting further studies.

**Patient concerns:**

A 51-year-old man presented to our outpatient clinic in October 2013 with complaints of difficulty to defecate and a change in stool characteristics. During digital rectal examination, a mass was palpated within 5 cm from the anal verge. Contrast-enhanced computed tomography revealed a 8.1 × 7.2-cm rectal mass with significant enhancement during the arterial phase.

**Diagnoses:**

A diagnosis of GIST was established after conducting needle biopsy and immunohistochemistry staining.

**Interventions:**

Imatinib therapy (400 mg/d, oral administration) was immediately started. When the patient achieved clinical CR (cCR), the oncologist recommended the patient to continue imatinib treatment.

**Outcomes:**

At 7 months after imatinib administration, the patient achieved cCR. As suggested by the oncologist, the patient continued to receive imatinib treatment after cCR. After 13 months, the patient spontaneously stopped imatinib. Finally, tumor recurrence was observed 7 months later.

**Lessons:**

Surgery remains the mainstay of treatment for advanced rectal GIST patients who achieve cCR after imatinib treatment. Close follow-up and continuous imatinib treatment are indicated in patients who cannot undergo surgery.

## 1. Introduction

Gastrointestinal stromal tumors (GISTs) are the most common mesenchymal tumors in the digestive tract, originating from interstitial cells of Cajal or their precursors and located in the stomach (60%) and the proximal end of the small intestine (30%).^[1]^ They contain gain-of-function mutations in c-KIT (75%–80%) or platelet-derived growth factor receptor alpha (PDGFRA) genes (5%–10%).^[2,3]^ In wild-type GISTs that lack c-KIT and PDGFRA mutations, loss-of-function mutations in the succinate dehydrogenase or loss of succinate dehydrogenase subunit B have been observed.

Current evidence suggests that rectal GISTs are relatively less common, with an incidence of 0.018 per 100,000.^[4]^ About three-quarters of patients are classified as high-risk patients with high metastatic rates to the liver, lung, and bone.^[5]^ Given the high degree of malignancy, early detection and treatment are essential to improve the prognosis. Before the introduction of imatinib, surgical resection was the only treatment for GIST, even for advanced lesions, which may be attributed to the poor response of GIST to conventional chemotherapy and radiotherapy.^[6,7]^ The recurrence rate after R0 resection is variable, with a 5-year survival rate ranging from 35% to 65%, depending on tumor size, mitosis, and tumor location.^[8]^ Importantly, imatinib, a tyrosine kinase inhibitor, can significantly suppress the malignant cells and improve the prognosis of most GIST patients. To the best of our knowledge, CR induced by imatinib has rarely been reported in the literature.

In this article, we introduced a case of rectal GIST who achieved CR after treatment with imatinib in our medical center and reviewed the related literature.

## 2. Case presentation

On October 5, 2013, a 51-year-old man was admitted to the hospital, with complaints of difficulty to defecate and a change in stool characteristics, but no hematochezia, melena, abdominal pain, bloating, nausea, and vomiting. The patient denied any prior medical history. The laboratory values were within the normal range. During digital rectal examination, a huge mass was palpated 5 cm from the anal verge.

A contrast-enhanced computed tomography (CT) scan of the abdomen showed a rectal mass (8.1 × 7.2 cm at its widest point) with obvious enhancement during the arterial phase with intact peripheral margins. No metastasis was found in the liver and lungs. A puncture biopsy was subsequently performed. Immunohistochemistry staining showed that the biopsied tissue was positive for clusters of differentiation CD34 and CD117. Mutations in exon 11 of the c-KIT gene were observed. Finally, the patient was diagnosed with rectal GIST (T3N0M0).

Given the patient’s refusal to undergo Mile’s resection and desire to preserve the anal sphincter, imatinib treatment (400 mg/d, oral) was immediately started. On the first abdominal CT scan performed 3 months after the initiation of treatment, the mass exhibited a partial response (PR). At 7 months, the CT scan showed local thickening of the lower rectum without enhancement. Then, magnetic resonance imaging showed similar findings. PET-CT showed no hypermetabolic lesion in the rectum, indicating clinical CR (cCR). At 10 months, a lower gastrointestinal endoscopic ultrasonography showed that the rectal wall structure was normal, and a biopsy was not required. The patient continued to take imatinib until 13 months after cCR. Seven months after discontinuing imatinib (20 months after cCR), tumor recurrence was observed. A contrast-enhanced CT scan showed that the intestinal space was full of soft tissue shadows. Biopsy of rectal mass showed soft tissue spindle cell carcinoma. Due to tumor progression, the patient was switched to sunitinib treatment (37.5 mg/d, oral). However, the patient was switched back to imatinib (600 mg/d, oral) after 11 months due to gastrointestinal bleeding. Six months later, the patient died of tumor-related causes.

## 3. Discussion

To improve our understanding of this rare case, studies from PubMed, Web of Science, and CNKI published from 2005 to 2021 were retrieved using the terms “Gastrointestinal Stromal Tumors” (Mesh) and “rectum” and “complete response” or “complete remission” and “imatinib.” Seven cases from 7 articles were included in this review.^[9–15]^ In addition to our case, 7 cases of rectal GIST were comprehensively analyzed in terms of clinicopathologic features, surgical treatment, imatinib dose, time to CR after initiation of imatinib therapy, and follow-up outcome (Table 1).

**Table 1:** A summary of all cases reported.

N	Author	Age (yr)	Gender	Tumor size (cm)	Tumor site	Mutation	Imatinib dose before surgery (mg/d)	Imatinib duration before CR	Treatment after CR	Follow-up interval	Recurrence
1	Salazar et al^9^	68	F	NA	Rectovaginal septum	Exon 11	400	12 m	Rectal resection and coloanal anastomosis	24 m	None
2	Hou et al^10^	72	M	7	Rectum submucosal extended into the prostate	NA	400	1.5 m	Segmental colorectal resection with sphincter-saving surgery	55.5 m	None
3	de Azevedo et al^11^	48	M	10	Pelvic	NA	400	15 m	Radical prostatectomy with sphincter preservation	15 m	None
4	Mandala et al^12^	57	M	14	Rectum extended in the gluteus region	Exon 11	400 then changed to 800	5 m	Abdominoperineal resection with permanent colostomy	18 m	None
5	Ciresa et al^13^	54	M	4.7	Rectum extended to the anal canal	NA	400	3 wk	Sphincter-saving surgery	NA	NA
6	Herawi et al^14^	51	M	1.7	Between the rectum and prostate extended off the surface of prostate	NA	400	NA	Local excision	48 m	None
7	Machlenkin et al^15^	NA	F	7	Lower two-third of rectum	NA	800	8 m	None	22 m	None
The present case	Wu et al	51	M	8.1	5 cm apart from the anus	Exon 11	400	7 m	Tyrosine kinase inhibitors continued for 30 mo	37 m	Local recurrence

In the RECIST criteria, cCR is defined as the absence of clinical, endoscopic, and imaging evidence of the tumor. For instance, in the present study, there was no evidence of the tumor on the CT scan or any hypermetabolic lesion on the PET scan after imatinib treatment. PR is defined as evidence of a decrease in size, necrosis, or cystic change of the lesion on radiological imaging. Pathologic CR (pCR) suggests no evidence of viable tumor cells.^[16]^

### 3.1. The clinical characteristics of the cases

#### 3.1.1. Clinicopathologic features, genetic investigation, and imatinib dose.

A total of 8 patients (6 males and 2 females) with a median age of 54 years at presentation (range, 48–72 years) were included in our review. The median tumor size was 7.0 cm (range, 1.7–14.0 cm). Three patients who underwent genetic testing presented mutations in exon 11. These 3 patients and 4 other patients initially received imatinib treatment of 400 mg/d. During treatment, for case 4, the imatinib dose was changed to 400 mg twice a day when his PET-CT showed one new FDG uptake focus around the tumor. Only case 7 took 800 mg of imatinib daily throughout the treatment. The median duration of imatinib treatment was 7 months (range, 0.75–15 months).

#### 3.1.2. Surgical treatment.

Six patients underwent surgical resection after imatinib treatment (see Table 1 for surgical methods), and pCR was achieved in all patients. Preservation of the anal sphincter was not feasible for only 1 patient (case 4) since the tumor was 14 × 10 cm large and extended to the gluteal muscle area. Accordingly, the patient underwent abdominoperineal resection and permanent colostomy. Two patients (cases 7 and 8) did not undergo surgery after achieving cCR.

#### 3.1.3. Postoperative management and clinical outcome.

After achieving cCR, only our patient continued to take 400 mg imatinib daily for 13 months, while other patients discontinued imatinib treatment. The average follow-up was 28.9 months (range, 0–55.5 months), and almost all patients were disease free. Only our patient (case 8) relapsed 20 months after cCR, 7 months after imatinib was halted.

### 3.2. Gene mutation status and medication guidance

The c-KIT mutation is reportedly present in 70% to 80% of GIST patients. The most common mutation is c-KIT exon 11, followed by c-KIT exon 9, while mutations in c-KIT exons 13, 14, 17, and 18 are rare.^[17,18]^ In recent years, the application of tyrosine kinase inhibitors targeting c-KIT and PDGFRA genes, such as imatinib, has achieved a breakthrough in the treatment of GIST, with 83% to 89% of patients with advanced GIST benefiting from imatinib.^[19,20]^ Given that the therapeutic effect of imatinib is directly related to the mutation status, it is necessary to conduct mutation analysis by immunohistochemistry before imatinib treatment. For tumors harboring kit exon 11 mutations, such as cases 1, 4, and 8, 400 mg imatinib once a day can cause a significant response. In case of tumor progression (for instance, case 4, where the PET-CT showed one new peritumor FDG uptake), the dose of imatinib was increased to 800mg. However, current evidence suggests that the prognosis of KIT exon 9 mutated intestinal GIST is poor, and 800 mg imatinib is recommended for initial administration every day.^[21]^ It is widely acknowledged that imatinib is the gold standard for patients with advanced or metastatic GIST disease. Sunitinib is recommended for patients with imatinib resistance or intolerance in an individualized treatment plan. Moreover, regorafenib has been approved for third-line therapy. Recently, avapritinib has been approved to treat patients with the resistant D842V mutation in the PDGFRA exon 18. Last but not the least, ripretinib has shown very promising activity in forth and further lines of therapy and has been approved in the United States.^[22]^

### 3.3. Efficacy evaluation of imatinib

During clinical practice, the efficacy of imatinib is usually evaluated by CT and magnetic resonance imaging; however, FDG-PET is considered a highly sensitive method for detecting early tumor response.^[23]^ Interestingly, Andtbacka et al^[24]^ pointed out that complete radiological and metabolic response was not completely consistent with pathological complete remission. In addition, pathologists may miss sporadic tumor cells in large lesions pretreated with imatinib. In this regard, KIT expression was missing in 20% of specimens after imatinib treatment.^[25]^ Most importantly, this may lead to false-negative pCR results.

### 3.4. Duration of imatinib

Data from all clinical trials showed that most patients who initially respond to imatinib eventually progress, indicating that imatinib rarely induces a complete pathological response. Accordingly, imatinib is not a cure for most patients^[25]^ but may act as a tumor inhibitor. There is currently no consensus on the continued use of imatinib in rectal GIST patients with cCR. The results of a randomized trial showed that imatinib interruption after 1 year was associated with a high risk of relapse, even in CR patients (treated with imatinib or surgery).^[26]^ The progression-free survival of patients with CR was equivalent to patients with PR or stable disease.^[26]^ In view of the above findings, further investigation evaluating the duration of imatinib administration is warranted. Imatinib may be used until intolerance or patient refusal. However, due to secondary resistance to imatinib, the duration of imatinib in most responders is not recommended to exceed 2 years.^[27]^ Alternatively, patients can switch to sunitinib. However, some imatinib-resistant patients were found to be nonresponsive to sunitinib.^[28]^

### 3.5. Perioperative treatment of imatinib

Based on findings from the literature, surgery remains the treatment of choice for patients with cCR. Close follow-up with imatinib treatment represents an alternative for patients where the anal sphincter cannot be preserved. In our case, due to the uncertainty of the scope of surgery and possible anal injury, no surgical treatment was performed, and imatinib treatment was continued with follow-ups scheduled.

Neoadjuvant treatment of imatinib may be an option for advanced rectal GIST.^[29]^ According to the literature, if technically feasible, transanal resection is recommended for non–high-risk rectal GISTs, while abdominoperineal resection should be considered only for high-risk or large non–high-risk rectal GISTs.^[30]^ Yang et al^[31]^ recommended that patients with tumors within 5 cm from the anus and <5 cm in size should undergo transanal surgery. Fortunately, neoadjuvant treatment of imatinib could facilitate the surgical procedure and minimize surgical injury, especially for low rectal GISTs.^[32,33]^ In our case, since the patient presented with a large tumor 5 cm within the anal verge, abdominoperineal surgery was indicated. Nonetheless, given the patient’s desire to preserve the anal sphincter, neoadjuvant treatment of imatinib was implemented. Our literature review showed that most patients avoided abdominoperineal resection, and anal sphincter function was preserved due to tumor shrinkage after imatinib therapy, which tremendously improved the quality of life. The interesting point about neoadjuvant treatment of imatinib is the timing of surgery. Surgical resection is generally recommended among the responders 6 to 12 months after the start of imatinib administration.^[27]^ Clinically, surgical resection is usually indicated when 2 consecutive imaging evaluations show stable disease.

Postoperative adjuvant imatinib therapy is widely used in GISTs with a high risk of recurrence, but the treatment course remains unclear. A randomized open-label phase 3 study SSGX VIII showed that 3 years of adjuvant therapy improved the progression-free survival and overall survival of GIST patients at high risk of recurrence.^[34]^ Moreover, a prospective, single-arm, phase 2 clinical trial recommended 5 years of adjuvant imatinib therapy for patients with resected primary GIST.^[35]^

## 4. Conclusion

Imatinib is reserved for patients with advanced high-risk rectal GIST as a neoadjuvant treatment or postoperative adjuvant treatment. Moreover, patients who achieved cCR after imatinib treatment can adopt nonoperative management or deferral of surgery strategy. Nonetheless, more information is needed in several aspects, such as identifying the real cCR and determining the optimal duration of imatinib treatment after cCR. Surgical resection should be recommended for rectal GIST patients with cCR after imatinib treatment. Close clinical monitoring and continued imatinib treatment are potential alternatives for patients where preservation of anal sphincter function is not feasible or patients unwilling to undergo surgery.

## Acknowledgments

This case report was prepared according to the CARE guidelines. Informed consent was obtained from the patient for publication of the case.
